# A rapid assessment of a community health worker pilot programme to improve the management of hypertension and diabetes in Emfuleni sub-district of Gauteng Province, South Africa

**DOI:** 10.3402/gha.v6i0.19228

**Published:** 2013-01-24

**Authors:** Tshipfuralo Ndou, Greer van Zyl, Salamina Hlahane, Jane Goudge

**Affiliations:** 1 Centre for Health Policy, School of Public Health, Faculty of Health Sciences, University of the Witwatersrand, Johannesburg, South Africa; 2 Department of Health and Social Development, Sedibeng District Health Service, Vereeniging, Gauteng Province, South Africa

**Keywords:** community health worker, chronic care, home visits, South Africa, hypertension, diabetes

## Abstract

**Background:**

Non-communicable diseases (NCD) and infectious chronic illnesses are recognised as significant contributing factors to the burden of disease globally, specifically in South Africa, yet clinical management is often poor. The involvement of community health workers (CHWs) in TB and HIV care in South Africa, and other low- and middle-income settings, suggests that they could make an important contribution in the management of NCDs.

**Objectives:**

Using a rapid assessment, this study examines the outcomes of a pilot CHW programme to improve the management of hypertension and diabetes in Gauteng province, South Africa.

**Methods:**

A record review compared outcomes of patients receiving home visits (n56) with a control group (n168) attending the clinic, matched, as far as possible, on age, gender, and condition. Focus group discussions and semi-structured interviews with CHWs, patients, district, clinic, and NGO staff were used to obtain descriptions of the functioning of the programme and patient experiences.

**Results:**

Despite the greater age and co-morbidity among those in the pilot programme, the findings suggest that control of hypertension was improved by CHW home visits in comparison to usual clinic care. However, too few doctor visits, insufficient monitoring of patient outcomes by clinic staff, and a poor procurement process for supplies required by the CHWs hampered the programme's activities.

**Conclusion:**

The role of CHWs in the management of hypertension should be given greater consideration, with larger studies being conducted to provide more robust evidence. Adequate training, supervision, and operational support will be required to ensure success of any CHW programme.

Management of chronic illnesses in low- and middle-income countries is often poor with health systems struggling with an outward flow of human resources and insufficient funds (1–3). There has been a re-emergence of community health worker (CHW) programmes in such settings to strengthen outreach activities and increase the number of health workers, in order to improve access to care (4). However, the focus of these programmes has predominately been on improving maternal and child-related health outcomes. Efforts to strengthen chronic disease management have focused primarily on HIV and TB treatment. In South Africa, CHWs have played a crucial role in the provision of effective HIV and TB care (from pre- and post-test counselling, administering treatment, adherence, to tracing defaulters) (5). The contribution of CHWs to infectious chronic disease care in South Africa suggests they could play an important role in non-communicable diseases (NCD) care.

This study provides a rapid assessment of a CHW pilot programme in Emfuleni sub-district of Gauteng, South Africa, that aimed to improve the management of hypertension and diabetes. A description of the programme and the research objectives in the next section are followed by the research methods, findings, and a conclusion discussing the relevance of the findings for national and international policy.

## The Kgatelopele community health worker programme

The Gauteng Provincial Department of Health South Africa in 2008 initiated the ‘Kgatelopele’ programme at one clinic in Emfuleni sub-district, in collaboration with Hands of Hope, a non-governmental organisation. The programme seeks to improve the management of hypertension and diabetes among patients by home delivery of medication and assessment of basic clinical indicators. Home visits by CHWs are intended to improve the accessibility, acceptability and affordability of health care, and strengthen the referral system between the community and health facilities. The CHWs provide social support and counselling to improve patient literacy and adherence, and to encourage appropriate visits to the PHC clinic. The clinic serves a population of 27,199, of whom 23,677 are un-insured, and hence use the public health sector. At the time of the study, patients enrolled on the Kgatelopele programme were visited once a month by one member of a team of six CHWs. A pharmacist packed a month's supply of medication for delivery to named patients. Patients are required to visit the clinic every 6 months for a physical examination by a doctor, who provides a renewed prescription. CHWs have attended a 14-week training course that focuses primarily on home-based care, other than providing skills in adherence counselling and health promotion, with a particular focus on chronic illnesses, including hypertension and diabetes. Other anticipated benefits of the programme included a reduction in clinic visits for elderly patients for whom there are physical challenges in attending the clinic, a reduction in transport cost for patients, and opportunities for CHWs to identify and refer other family members in need of health care. Hypertensive and diabetic patients not in the pilot programme attend the clinic on a monthly basis.

The objectives of the study are to, first, provide a comparison of treatment outcomes to give some insights into how extension of care into the community may be developing; second, to describe the operational challenges of the programme; and finally, to draw lessons relevant to CHW programmes providing NCD care in other settings, both within South Africa and other low- and middle-income countries.

## Methods

We were asked by the Sedibeng district manager to conduct a rapid evaluation of the pilot CHW programme. The evaluation used a retrospective case study approach, employing both qualitative and quantitative methods. The study population consisted of individuals registered in the Kgatelopele programme (56 patients) in Emfuleni sub-district, Gauteng, as well as patients receiving usual clinic care at the same clinic. Interviews were conducted with 20 Kgatelopele patients on their experience of the programme. Seven key informants (clinic, NGO, and district staff) were interviewed to obtain information on the training of CHW, supervision, procurement, and referral processes in addition to other elements of the programme operation and its challenges. Two focus group discussions were held with two groups of six CHWs to obtain a description of practical day-to-day functioning of the pilot and its challenges.

A record review was undertaken, using a pre-designed record review form, of patients on the Kgatelopele programme and a control group comprising three clinic patients matched for condition, age, and gender for every Kgatelopele patient. The Kgatelopele programme enrolled stable patients, and a clinic patient was deemed a suitable match if, where possible, the person was within 5 years of age the Kgatelopele patient.With 56 patients in the intervention group and three-fold more controls, we would be able to detect a difference between groups if hypertension was controlled in 40% of intervention group and in 20% of controls, with 80% power (control defined as having a normal blood pressure (BP) for at least >40% of clinic visits). There is little gain in power with having more than three-fold the number of participants in the control arm.

The record review collected data for the year prior to the study on a number of variables, including age, sex, number of home visits, monthly measures of BP and blood glucose, doctor reviews, and clinic attendance. For this study, we took 110/60–130/85 mmHG as an acceptable range for controlled hypertension. We used 3.6–5.8 mmol/l as an acceptable range for controlled diabetes. The record review data were analysed by comparing the BP levels and glucose level (Hgt) (using a random sample) of patients on programme and comparison group.

Thematic analysis was conducted on interview and focus group discussion transcripts, identifying data on *a priori* and emergent themes. Respondent views that were divergent from common perspectives were explored further to ensure opposing views were retained within the analysis. Ethical approval was granted by the University of the Witwatersrand Committee for Research on Human Subjects. Informed consent was given by all respondents.

## Results

The sample consisted of 56 patients on the outreach programme and 168 clinic patients, with slightly more female patients than males in both groups (Table 1). Despite attempts to match patients on the programme with those at the clinic, there were important differences between the two groups.

**Table 1:** Description of the sample and the comparison group

	Kgatelopele patients	Clinic patients
Number of patients	56	168
Female (*n*)	69.6 (39)	69.9 (117)
Mean age (range)	75 (54–96)	69 (51–92)
Patients with co-morbidities (*n*)	39.3 (22)	25.0 (42)
Months on treatment recorded in file (range)*	9 (1–20)	6 (2–13)
Months on Kgatelopele programme	8 (2–18)	N/A

* When treatment was interrupted or the patient's record could not be found, a new record was started. Hence, the relatively short period on treatment is unlikely to be accurate.

The mean age of those on the programme was 75 years (range 54–96), and 69 years (range 51–92) in the clinic group. Nearly 40% of the programme patients had both hypertension and diabetes, in comparison to 25% in the clinic group. The mean number of months on treatment was nine (range 1–20) compared to 6 months (range 2–13) for clinic patients, and the mean number of months since enrolment on the programme was eight (range 2–18).

### Comparison of outcomes

For patients with hypertension, the condition was controlled for a higher proportion of patients on the Kgatelopele programme in comparison with those attending the clinic (Table 2). 21.4% of Kgatelopele patients (12/56) were controlled at >40% of health checks in comparison to 13.1% of clinic patients (22/168). In contrast, diabetes was better controlled among clinic patients, with 26.1% (11/42) controlled for >40% of health checks compared to only 9.1% (2/22) of the Kgatelopele patients.

**Table 2:** Percentage of patients with controlled hypertension and diabetes at health checks in the past year in the programme and control groups (*n*)

Percentage of health checks in the previous year at which the condition was controlled	Hypertension patients			Diabetes patients			BP control for patients with both conditions		
		
	Kgatelopele	Clinic	*P*	Kgatelopele	Clinic	*P*	Kgatelopele	Clinic	*P*
0–40	78.6 (44)	86.9 (146)	0.13	90.9 (20)	73.8 (31)	0.11	72.7 (16)	95.2 (40)	0.01
41–100	21.4 (12)	13.1 (22)		9.1 (2)	26.2 (11)		27.3 (6)	4.8 (2)	
									
Total	100% (56)	100% (168)		100% (22)	100% (42)		100 (22)	100 (42)	

Health checks may have occurred either on a home visit or a 6-monthly clinic review.

If we consider only those *with both conditions*, hypertension was controlled amongst a considerably higher proportion of Kgatelopele patients (27.3%; 6/22) >40% of health checks in comparison with 4.8% of clinic patients (2/42).^1^

### Demand-side issues

Study participants stated that the Kgatelopele programme had a positive impact on access to care: “Kgatelopele is helping many people and is saving many people's lives. So people in this area are getting help from it and they are happy with the program” (KII). In particular, the programme assisted elderly patients who had difficulties travelling to the clinic:

With the problem of taxis and being old, this programme is helping by delivering my medication at home. (Patient)

Patients were also satisfied with the CHW counselling and health information:

The care giver who comes here always gives me advice on how to avoid high BP and sugar level. She told me that if I don't follow the advice it might cause me stroke. (Patient)

A variety of explanations were given for the overall poor levels of control of both conditions. Some patients explained they discontinued their medication because of side effects: staff confirmed this.

Patients are defaulting. They are not taking medication as they are directed. Medications are packed with written instructions but then the patient will not take some medication; they will choose (which pills to take). (KII – Clinic nurse)

The elderly respondents reported difficulty in attending the 6 monthly review (by a doctor) at the clinic due to the inability to walk to the public transport, or funds to pay for public transport. One key informant also reported: “Patients do not want to go see a nurse. When we tell the patients they must come to the clinic they say ‘You said I must come to see a doctor; so where is the doctor, not a nurse again’”. As a result, the patients’ prescriptions are not renewed and treatment is missed.

### Supply-side issues

An important benefit of the programme was the reduction in the patient load at the clinic:

It is good programme because our clinics are flooded with patients. Some of the patients really don't need to be at the clinic. (KII)

There were different views as to the criteria for enrolling patients on the programme, with some respondents stating that the programme should be for all chronic disease patients. Other respondents were of the view that younger, more mobile patients should come to the clinic, as they are often not at home when the CHW make visits.

I don't think she (the doctor) understands what is happening really, even people who are very well who can come to the clinic, but I took them on the side and said no you are not going to be on the program, we are taking care of people who cannot come to the clinic, those who must hire the transport to the clinic because they are very sick. (KII)

However, an insufficient number of visits to the clinic by the rotating doctor increased the number of visits required to renew a prescription. The doctor was not available for the patient review as scheduled in 14% of cases.

The first challenge is the availability of the doctor and this is the serious one. When I say the availability of the doctor I mean like now the last time she came here was 12th of August till today (19 October). So the patients who were supposed to be reviewed are not getting medication. (KII – District staff)

In addition, the insufficient number of doctor visits limited clinical supervision available for nurses.

The interview data provided a limited explanation as to the differential effects on control of hypertension and diabetes. CHWs and NGO staff complained of shortages of glucose strips, which are likely to have hindered monthly monitoring of blood sugar levels, and efforts at self-management by patients. Lack of materials, a key to barrier effective functioning of the programme, was caused by a lack of dedicated funding and unclear procurement processes and responsibilities:

The [provincial Department of Health] gave us the bags and there's BP machine, sugar strips and everything but when those things are finished we (NGO) have to find the money (for materials such as glucose strips). (KII – NGO staff)

No regular meetings among the NGO, clinic staff, and district staff were held to discuss issues such as shortages of CHW equipment, or funding constraints to purchase new supplies.

CHW records provided descriptive data on activities, such as the number of patients seen, patients’ health complaints and dates for next visit. The information collected by the CHW was reported to supervisors at the NGO, who did not see it as their responsibility to intervene should a patient have poor clinical outcomes. Clinic nurses did not regularly examine the CHW records. As a result, they did not identify, or take action to assist patients that required intervention or referral.

I don't get time to communicate or sit with the NGO manager and discuss the work of CHWs or which patients were added. They could at least give me copy of papers where CHWs record test results for patients. (KII)

## Discussion

Systematic reviews have provided evidence of the efficacy of CHW programmes with respect to certain health service outcomes (such as immunisation, uptake of breast feeding, TB treatment compliance), in a range of different low- and middle-income settings (6). However, there is little evidence on the effectiveness of their role in management of hypertension and diabetes. The comparison of the outcomes of this pilot programme with usual care is limited by the differences in age and co-morbidity between the two groups of patients. Further limitations include that BP was measured by CHW rather than nurses (electronic devices were used), the range for controlled hypertension was lower than the internationally accepted range, and that random glucose estimation was used for assessing control of diabetes rather than a fasting or HbA1C test. However, despite these limitations, the findings suggest that hypertension is better controlled amongst patients in the pilot programme.

Despite concerns of adherence and problems with doctor availability, these findings suggest home delivery of medication and monitoring by CHWs (rather than nurses) did not worsen control of hypertension; instead the benefit of not having to travel to the clinic, as well as perhaps the care provided in the home setting, led to better control. The findings of this study suggest that the role of CHW in the management of hypertension should be given greater consideration, with larger studies being conducted to provide more robust evidence.

However, the same was not true for diabetes, where the condition was better managed among clinic patients. Diabetes was controlled for 26% of patients at the clinic in comparison to 9% of the Kgatelopele patients. The explanation for this was unclear from the interviews. It may be due to insufficient training of CHW on assisting patients to manage diabetes (mentioned in the description of the programme), a condition that requires more active self-management by patients (7), or the irregular supply of glucose strips, preventing the CHWs from monitoring glucose levels.

Although the level of control for either condition was far from ideal, the findings were not dissimilar to other South African studies. (The level at which BP is deemed controlled varies across research studies, reducing ability to draw comparisons. The level used in this study (130/85 mmHg) is lower than the international and South African standard (140/90 mmHg). Edwards et al. (8) assessed hypertension control levels of about 12,000 South African hypertensive private patients with medical aid insurance of whom 34.7% had BP <140/90 mmHg. A study of 9,133 patients attending 680 private practices throughout the country found 53% of patients with controlled hypertension (<140/90 mmHg) (9). Both studies suggest better management of hypertension in comparison to the Kgatelopele programme, possibly due to provision of care by private general practitioners, and a higher cut-off of 140/90 mmHg rather than 130/80 mmHg used in this study. However, a population survey at the rural health and demographic surveillance site in Mpumalanga of pre-dominantly public health service users, found only a small proportion (∼9%) of people with hypertension had their BP successfully controlled with medication (Xavier Gomez-Olive – personal communication). In a study at Hlabisa district hospital, Kwa Zulu Natal, of 164 patients with both diabetes and hypertension, only 20% achieved a target BP of <130/85 mmHg (10), and acceptable glycaemic control (HbA1c < 2% above normal population range) was found in only 15.7% of subjects (95% confidence interval).

International evidence has shown that CHW programmes often do not yield the expected outcomes because of insufficient training and skills, and inadequate support from the health system (both in terms of clinic staff and referrals to high levels) (11). The findings from this study suggest similar conclusions. Higher quality and more relevant training for CHW (which previously focused on TB and HIV) are needed as such training has shown to be successful in other low- and middle-income settings (12).

Lack of support from the health system was evident in that the CHWs did not report their monthly clinical assessments of patients to the nurses. As a result, the information that was collected did not trigger an intervention for patients whose condition was not controlled. Moreover, too few doctor visits to the clinic prevented renewal of patient prescriptions and hence limited clinical oversight. Furthermore, poor procurement processes for required materials hampered programme activities. In addition, poverty and the associated social determinants of health, often result in barriers to care that CHW are unable to resolve without intervention from other sectors such as social welfare (13). Again this is reflected in this study with patients experiencing considerable difficulty in attending the clinic for the doctor's review.

South Africa's antiretroviral treatment programme, the largest worldwide with recent notable increases in coverage, has generated substantial knowledge on how to improve adherence, tracing defaulters, and enabling patient participation through treatment literacy and patient support groups (5). It is important that this knowledge and experience is translated into the management of other chronic conditions, such as hypertension and diabetes, in this and similar outreach programmes. Other low- and middle-income countries have begun to integrate HIV care with hypertension and diabetes (14), or provide an integrated chronic care service (15). The findings of this rapid assessment of a pilot programme suggest that CHW can make a useful contribution to the management of chronic conditions. However, to ensure greater success, more effort is required to ensure there are effective procurement processes, communication between clinic and outreach staff, as well as better clinical supervision than was demonstrated in this pilot.

## Conclusion

Outcomes of this pilot programme suggest that home visits and delivery of medication by CHWs can lead to improved control of hypertension in comparison to patients attending the public clinics on a monthly basis. However, this programme did not meet CHW training and supervision needs, or ensure effective procurement processes. Further efforts are required in this regard for CHW programmes to contribute successfully to improved management of NCD.

## Footnotes

1 All diabetic patients also had hypertension.
